# Multidrug Intrinsic Resistance Factors in *Staphylococcus aureus* Identified by Profiling Fitness within High-Diversity Transposon Libraries

**DOI:** 10.1128/mBio.00950-16

**Published:** 2016-08-16

**Authors:** Mithila Rajagopal, Melissa J. Martin, Marina Santiago, Wonsik Lee, Veronica N. Kos, Tim Meredith, Michael S. Gilmore, Suzanne Walker

**Affiliations:** a Department of Microbiology and Immunobiology, Harvard Medical School, Boston, Massachusetts, USA; b Department of Chemistry and Chemical Biology, Harvard University, Cambridge, Massachusetts, USA; c Department of Ophthalmology, Harvard Medical School, Massachusetts Eye and Ear Infirmary, Boston, Massachusetts, USA

## Abstract

*Staphylococcus aureus* is a leading cause of life-threatening infections worldwide. The MIC of an antibiotic against *S. aureus*, as well as other microbes, is determined by the affinity of the antibiotic for its target in addition to a complex interplay of many other cellular factors. Identifying nontarget factors impacting resistance to multiple antibiotics could inform the design of new compounds and lead to more-effective antimicrobial strategies. We examined large collections of transposon insertion mutants in *S. aureus* using transposon sequencing (Tn-Seq) to detect transposon mutants with reduced fitness in the presence of six clinically important antibiotics—ciprofloxacin, daptomycin, gentamicin, linezolid, oxacillin, and vancomycin. This approach allowed us to assess the relative fitness of many mutants simultaneously within these libraries. We identified pathways/genes previously known to be involved in resistance to individual antibiotics, including *graRS* and *vraFG* (*graRS/vraFG*), *mprF*, and *fmtA*, validating the approach, and found several to be important across multiple classes of antibiotics. We also identified two new, previously uncharacterized genes, *SAOUHSC_01025* and *SAOUHSC_01050*, encoding polytopic membrane proteins, as important in limiting the effectiveness of multiple antibiotics. Machine learning identified similarities in the fitness profiles of *graXRS/vraFG*, *SAOUHSC_01025*, and *SAOUHSC_01050* mutants upon antibiotic treatment, connecting these genes of unknown function to modulation of crucial cell envelope properties. Therapeutic strategies that combine a known antibiotic with a compound that targets these or other intrinsic resistance factors may be of value for enhancing the activity of existing antibiotics for treating otherwise-resistant *S. aureus* strains*.*

## IMPORTANCE

Bacterial resistance to every major class of antibiotics has emerged, and we are entering a “post-antibiotic era” where relatively minor infections can lead to serious complications or even death. The utility of an antibiotic for a specific pathogen is limited by both intrinsic and acquired factors. Identifying the repertoire of intrinsic resistance factors of an antibiotic for *Staphylococcus aureus*, a leading cause of community- and hospital-acquired infections, would inform the design of new drugs as well as the identification of compounds that enhance the activity of existing drugs. To identify factors that limit the activity of antibiotics against *S. aureus*, we used Tn-Seq to simultaneously assess fitness of transposon mutants in every nonessential gene in the presence of six clinically important antibiotics. This work provides an efficient approach for identifying promising targets for drugs that can enhance susceptibility or restore sensitivity to existing antibiotics.

## INTRODUCTION

*Staphylococcus aureus* is a Gram-positive pathogen with a remarkable ability to withstand antibiotics and evade the human immune system. Many factors, both intrinsic and acquired, have been shown to contribute to its ability to survive specific antibiotic stress. For example, methicillin-resistant *S. aureus* (MRSA) strains have acquired the mobile staphylococcal cassette chromosome *mec* element (*SCCmec*), encoding a transpeptidase, PBP2A, which is naturally resistant to β-lactams, enabling the organism to make cross-linked peptidoglycan when the native transpeptidases are inactivated by the β-lactams (1–3). Irrespective of its methicillin susceptibility status, *S. aureus* possesses numerous intrinsic factors that also limit the effectiveness of specific antibiotics (4). In contrast to acquired resistance factors like PBP2A, intrinsic resistance factors typically play additional roles in normal microbial physiology. For example, MprF, which modulates cell membrane charge, was initially identified in *Staphylococcus xylosus* as a gene that, when inactivated, increased susceptibility to the cationic peptide gallidermin (5). The activity of MprF is now known to be important for protection from other cationic antimicrobial peptides and daptomycin (6–8). TarO, which catalyzes the first step in the wall teichoic acid biosynthetic pathway (9), contributes to β-lactam resistance in MRSA, and its deletion results in cell division defects and mislocalization of cell wall biosynthetic machinery (10–12). Effective pharmacological inhibition of TarO in MRSA restores full sensitivity to β-lactams even though PBP2A is present (10, 12), highlighting the potential to mitigate antibiotic resistance by targeting intrinsic resistance factors. The most attractive candidates for targeting are those factors that hinder the activity of multiple classes of antibiotics. To identify such candidates, as well as additional factors contributing to the resistance of specific individual antibiotics, we used the massively parallel approach of transposon sequencing (Tn-Seq) (13–15) to examine large pools of *S. aureus* transposon mutants for fitness defects upon exposure to multiple classes of antibiotics.

Tn-Seq involves creating large transposon libraries, sequencing the transposon insertion sites with next-generation sequencing, and mapping the sequence reads to a reference genome (13, 15). This technique can be used to identify genes that contribute to fitness in a particular environment or under a particular set of growth conditions because reads mapping to these genes would be depleted compared to reads in a control. Depletion of reads in a gene implies that the mutants have reduced fitness under the test conditions. Tn-Seq can also be used to identify those insertion mutants that are highly represented in the mutant pool, indicating that inactivation of those genes increases fitness under the tested condition.

**FIG 1:**
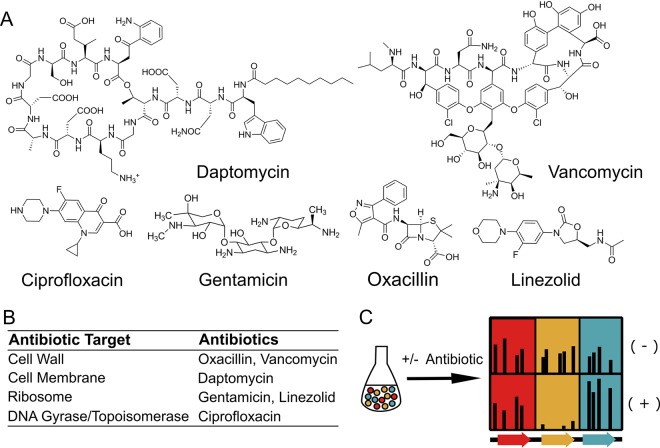
Intrinsic resistance factors that contribute to antibiotic resistance can be identified by Tn-Seq. (A) The structures of the six different antibiotics used in the Tn-Seq experiments are shown. (B) The targets of the six antibiotics are shown. (C) A pooled transposon insertion library was grown with or without antibiotic and subjected to Tn-Seq to quantify the number of sequencing reads that map to each insertion location. The black lines in the columns corresponding to the three genes represent the number of reads mapping to a particular insertion location. In this example, the red gene had similar numbers of reads in the treated and untreated samples. Therefore, inactivation of this gene does not have an effect on antibiotic susceptibility. The orange gene had a lower number of reads in the treated sample than in the untreated control. Inactivation of this gene decreases bacterial fitness in the presence of the tested antibiotic. Genes of this type are known as intrinsic resistance factors. Finally, the blue gene had a higher number of reads in the treated sample than in the untreated control. Inactivation of this gene increases bacterial fitness in the presence of the test antibiotic.

Tn-Seq has been used previously to identify antibiotic resistance factors for other organisms (16–18) but has not been used to compare multiple antibiotic classes in *Staphylococcus aureus*. We have performed Tn-Seq analysis using transposon libraries treated with six different antibiotics to identify genes with significantly fewer mapped reads than were seen with an untreated control. These genes, which we refer to as intrinsic resistance factors, render the bacteria more sensitive to the antibiotic tested when inactivated. The six antibiotics used in this study, ciprofloxacin, linezolid, gentamicin, oxacillin, vancomycin, and daptomycin, were chosen as clinically relevant representatives of antibiotics that target major pathways: DNA synthesis, protein synthesis, cell wall synthesis, and membrane stability (Fig. 1A and B) (19–25). In addition to previously identified factors, we have identified two hitherto-uncharacterized factors as important intrinsic resistance factors for multiple antibiotics.

## RESULTS AND DISCUSSION

### Experimental approach and data analysis.

Two different transposon libraries constructed in methicillin-susceptible *S. aureus* (MSSA) strain HG003 were used in initial screens for mutants exhibiting either enhanced resistance or enhanced susceptibility to an antibiotic (14, 26). The first transposon library was made by transformation with a temperature-sensitive plasmid and contained insertions in 71,000 unique sites (26). The second library was made using a phage-based transposition system and included transposon insertions in 126,040 unique sites (14). To identify mutants that exhibit fitness defects that are independent of the growth condition (and therefore likely to be of value *in vivo*), the libraries were treated with six antibiotics at concentrations below the MIC of the antibiotics and grown in different media for various numbers of generations (see Materials and Methods).

Sample preparation and Tn-Seq analysis to determine the location of the transposon insertions were performed as described previously (14, 26, 27). Subsequently, the number of reads mapping to a gene under experimental antibiotic treatment conditions was compared to the number in the untreated control to calculate the fold change in the number of reads mapping to each gene (Fig. 1C) as a surrogate measure of the representation of each mutant in the pool. Data were analyzed as previously described (14, 17), with one additional step: before comparing the number of reads/gene using the Mann-Whitney *U* test, the experimental condition (antibiotic treatment) was normalized to that of the untreated control using simulation-based resampling to minimize differences between the two conditions (28, 29). After all experiments for the two libraries were analyzed independently, *P* values and depletion/enrichment ratios for each gene in the presence of an antibiotic treatment were combined, using Fisher’s method for *P* values and the geometric mean of fold changes in the number of reads mapping to each gene.

Because antibiotics with differing mechanisms of action exert different degrees of selective pressure on the bacterial population at fractional MIC levels, it was necessary to tailor the data analysis approach in a way that enabled comparison across different antibiotic treatments. We therefore adjusted the cutoff value for the fold change in the number of mapped reads/gene for the treated samples relative to the controls to obtain similar numbers of hits for each antibiotic. Only genes corresponding to a *P* value of <0.05 were considered. We used a sliding cutoff value for the number of reads/gene that resulted in a maximum of 20 genes being identified as hits for each antibiotic. This sliding fold change cutoff value ranged from 10-fold (0.1 to 10) for ciprofloxacin to 55-fold for oxacillin. The top genes contributing to susceptibility/resistance for each antibiotic included genes with fewer as well as more reads mapping to them in the treated sample than in the control. The former represent intrinsic resistance factors or impediments to antibiotic inhibition of *S. aureus*. The latter are also of interest as they provide information on how antibiotic resistance can arise via gene inactivation.

### Intrinsic factors that decrease or increase susceptibility to antibiotics.

Of the genes implicated in antibiotic resistance via the analysis described above, 80 were unique (see Table S1 in the supplemental material). Among the 20 genes in which transposon insertion resulted in the greatest change with respect to antibiotic susceptibility/resistance, we identified few or none that, when mutated, provided a fitness advantage upon exposure to ciprofloxacin, daptomycin, linezolid, oxacillin, and vancomycin, with the notable exception of gentamicin. For gentamicin, half of the mutants exhibited a fitness advantage (Table 1; see also Fig. S1 in the supplemental material). All of these genes, the absence of which enhanced fitness in the presence of fractional MIC levels of gentamicin, occur in the oxidative phosphorylation pathway. It is known that gentamicin and other aminoglycosides rely on the membrane potential to gain entry into cells (30, 31); disrupting genes in the oxidative phosphorylation pathway therefore limits cellular penetration.

**TABLE 1:**
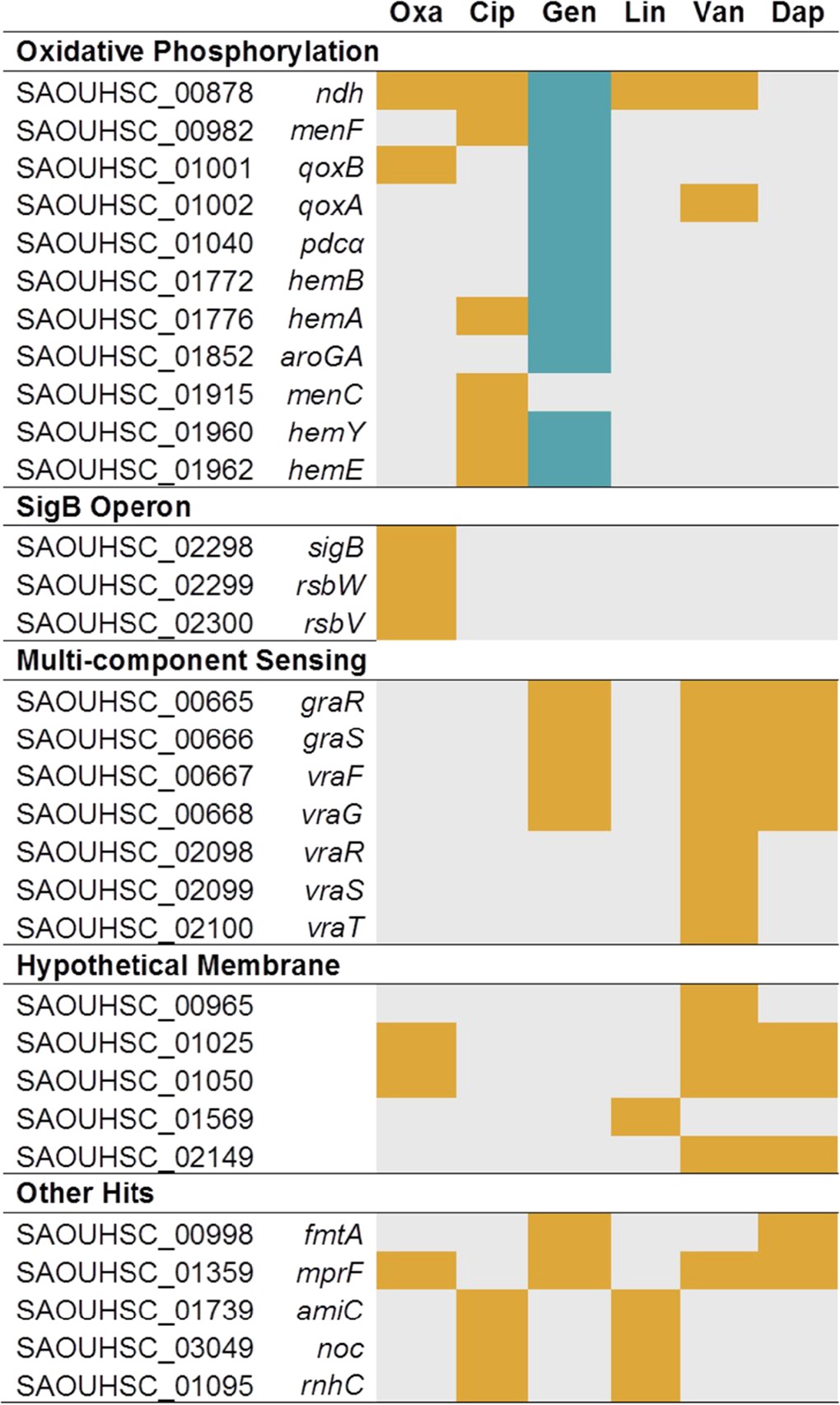
Treatment of a pooled transposon library with six different antibiotics identified genes that contribute to fitness under antibiotic stress conditions^a^

a Orange rectangles indicate genes for which the numbers of reads due to transposon insertions were substantially lower than in the control, whereas blue rectangles indicate genes for which the numbers of reads due to transposon insertions were substantially higher than in the control. Gray rectangles indicate those genes which were not identified among the top 20 most affected genes. The top 20 most affected genes for each antibiotic were identified, and a subset of the 80 unique genes is shown here. The complete list is shown in Table S1 in the supplemental material. oxa, oxacillin; cip, ciprofloxacin; gen, gentamicin; lin, linezolid; van, vancomycin; dap, daptomycin.

This analysis also identified many other genes for which a contribution to resistance/susceptibility to an antibiotic had been observed previously. For example, *sigB* was among the hits identified with oxacillin treatment. Reads mapping to this gene, and to other components involved in the alternative sigma factor pathway, i.e., *rsbV* and *rsbW*, were significantly depleted after growth with oxacillin (>100-fold depletion in reads/gene for all three genes) (Table 1). It has been shown that overexpressing SigB causes cells to have thicker cell walls, increased transcript levels for penicillin-binding proteins, and elevated MICs to β-lactams and that deletion of *sigB* renders resistant *S. aureus* strains more sensitive to oxacillin (32, 33). Reads for *pbp4*, which encodes a penicillin-binding protein involved in secondary cross-linking of peptidoglycan and β-lactam resistance (34–36), were also found to be depleted under conditions of oxacillin treatment. Similarly, reads mapping to all three genes of the *vraTSR* operon, which encodes a multicomponent sensing (MCS) system that regulates the cell wall stress stimulon (37–41), were substantially depleted in the presence of vancomycin. *norA*, which encodes an efflux pump that is known to be involved in ciprofloxacin resistance (42, 43), was also identified as an important factor under conditions of ciprofloxacin treatment. In addition to these and other known intrinsic resistance factors, we identified 13 hypothetical genes that are important for resistance to these six antibiotics (see Table S1 in the supplemental material).

While no inactivated gene altered susceptibility to all six antibiotics, we did identify 21 genes that were hits with two or more antibiotics, and 8 of these were hits with more than two antibiotics (see Table S1 in the supplemental material). These 8 genes included *mprF*, *ndh*, *fmtA*, components of the *graRS* and *vraFG* (*graRS/vraFG*) multicomponent sensing system, and two genes of unknown function, *SAOUHSC_01025* and *SAOUHSC_01050.*

**FIG 2:**
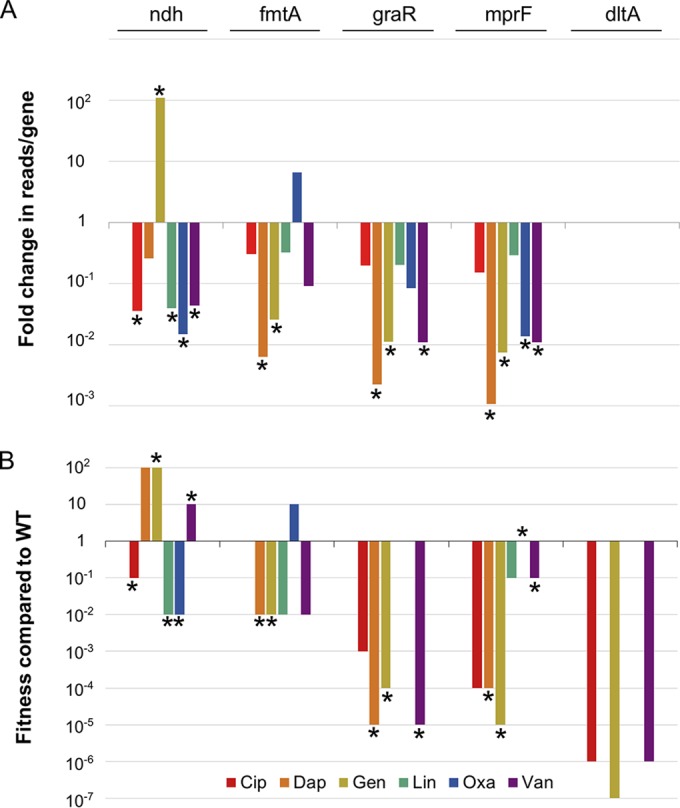
Tn-Seq results were validated by testing mutant fitness in spot dilution assays. Tn-Seq results and validation for selected genes are shown. *ndh* encodes an NADH dehydrogenase involved in oxidative phosphorylation; *fmtA* is a cell surface protein of undetermined function; *graR* is a member of a multicomponent sensing system (MCS); *mprF* and *dltA* are members of the regulon of this MCS. Asterisks indicate those conditions under which the pertinent gene was among the top 20 hits for that antibiotic. (A) Bar graph depicting fold change in reads per gene relative to the untreated control for each of the six antibiotics. As very few insertions in *dltA* were present in the untreated control, changes in fitness could not be detected by Tn-Seq. (B) Bar graph depicting fitness of mutant strains in which the indicated genes are inactivated compared to that of the WT. Fitness was assessed by spotting 10-fold dilutions of WT and mutant strains on antibiotic plates and comparing the highest dilutions that resulted in growth (see Materials and Methods). Because the data are shown on a logarithmic scale, the lack of a bar indicates that no change (fold change value of 1) was observed for that mutant under the relevant treatment condition. Fitness assessed by spot dilution validated the Tn-Seq results for the list of the top 20, with the exception of *ndh* with vancomycin treatment. Strain backgrounds were USA300 LAC JE2 for the *ndh*, *fmtA*, and *mprF* transposon mutants and Newman for the *graR* and *dltA* deletion mutants.

Our ability to detect numerous previously identified resistance factors using this massively parallel fitness profiling approach served as a validation of the method. To further confirm the results and to determine whether these resistance factors were also important in other *S. aureus* strains, we examined the fitness of mutants in genes identified as hits against all six antibiotics under two or more sets of conditions using an agar spot dilution assay (Fig. 2; see also Fig. S1 in the supplemental material). Mutants were chosen based on whether they were identified as hits under two or more sets of conditions. In general, the agreement between Tn-Seq results and the spot dilution assay results was excellent. Given that the spot dilutions did not involve competition between thousands of mutants and that the assays were performed at a single concentration (chosen so that wild-type [WT] growth would be relatively unaffected; see Materials and Methods) and involved mutants from different genetic backgrounds (Newman or USA300 instead of HG003), the high validation rate is remarkable and supports the choice of the approach described here.

### Intrinsic factors that impact multiple classes of antibiotics.

Of the eight genes found to impact multiple classes of antibiotics, *ndh* (NADH dehydrogenase) is the only one that, when inactivated, promotes resistance to some antibiotics while promoting sensitization to others. Ndh is a component of the electron transport chain. The electron transport chain creates a membrane potential, which is required for penetration of gentamicin through the cell membrane (44). We show here that, in addition to conferring resistance to gentamicin when inactivated, *ndh* is an intrinsic resistance factor for oxacillin, linezolid, and ciprofloxacin. *ndh* is in the same pathway as several genes that are commonly found to be inactivated in small colony variants (SCVs), a phenotype correlated with persistent infections that are resistant to β-lactams as well as aminoglycosides (45–48). Our data confirmed the importance of the oxidative phosphorylation pathway in antibiotic resistance, identified new genes of importance, and suggest that a better understanding of how *S. aureus* modulates this system could increase our understanding of antibiotic resistance.

FmtA is a cell surface protein of uncertain function. It was identified as a factor involved in methicillin resistance and has since been proposed to act as a carboxypeptidase and a teichoic acid d-ala esterase (49–51). We did not find that inactivation of *fmtA* resulted in increased sensitivity to oxacillin in the Tn-Seq analysis or in the agar spot dilution assay. In fact, in these tests, fitness was enhanced compared to that of other mutants under conditions of oxacillin selection. However, we did observe increased sensitivity of *fmtA* mutants to daptomycin and vancomycin. The impact of *fmtA* mutations showed that *fmtA* met the threshold for inclusion among the top 20 genes affecting daptomycin resistance but was not among the top 20 for vancomycin selection. Nevertheless, these data support the idea of an important role for FmtA in withstanding cell envelope stress for at least some classes of antibiotics (52).

MprF catalyzes formation of lyslyphosphatidylglycerol, a membrane modification that confers protection against cationic antibiotics, which are repelled by the increased cell surface positive charge (6, 8, 53, 54). MprF is also known to contribute to methicillin resistance in MRSA strains (55), and point mutations increasing the activity of MprF are a mechanism for daptomycin resistance (7, 56, 57). In our studies, *mprF* was found to be an intrinsic resistance factor for all antibiotics tested, although it occurred among the top 20 for only four of the six (Table 1 and Fig. 2A). Enhanced susceptibility of *mprF* mutants to these four antibiotics was also found in the agar spot dilution assay performed using mutants in the USA300 (MRSA) background (Fig. 2B) (58). Since *mprF* inactivation potently promotes sensitization to daptoymcin, vancomycin, and ciprofloxacin, which are not rich in positive charges, the positively charged product of MprF, lysylphosphatidylglycerol, likely plays biophysical roles in membrane stability or organization. This has been suggested previously for daptomycin (59, 60).

### GraRS/VraFG is the most important MCS across tested antibiotics.

Multicomponent sensing systems (MCSs) allow bacteria to sense and respond to their environments. These systems typically include a membrane-anchored extracellular sensory domain fused to an intracellular kinase domain and a separate, cytosolic response regulator, but they can also include additional elements. A stimulus sensed by the sensory domain results in a change in the phosphorylation state of the response regulator, which in turn modulates the expression of downstream targets (61). *S. aureus* contains many multicomponent sensing systems, and we identified multiple components of three of these systems, *agrABCD*, *vraTSR*, and *graXRS/vraFG*, among the top hits under conditions of selection with at least one antibiotic (Table 1; see also Table S1 in the supplemental material).

*AgrABCD* is involved in quorum sensing and regulation of virulence factors and autolysin expression (62–64). Sequencing reads from transposon insertions mapping to components *agrA*, *agrB*, and *agrD* were depleted under conditions of oxacillin or daptomycin treatment, suggesting that factors regulated by this MCS are involved in the response to these antibiotics, possibly due to its regulation of the autolysin *lytM* and the penicillin-binding proteins (64–66). While reads mapping to *agrC* were also depleted under these treatment conditions, *agrC* did not meet the threshold for inclusion in the top 20 hits. The *vraTSR* system is known for its crucial role in withstanding vancomycin treatment (38), and all three components were among the top genes identified under conditions of vancomycin treatment. This sensing system regulates expression of cell wall biosynthetic genes, and it has also been implicated in β-lactam resistance (37, 39, 67). Although reads mapping to these genes were also depleted under conditions of oxacillin treatment, they were not depleted enough to be included among the top 20 genes contributing to oxacillin resistance.

The single most important MCS across all the six antibiotics tested is *graXRS/vraFG*. Four components of this system met our cutoffs under conditions of gentamicin, daptomycin, and vancomycin treatment (Table 1). Moreover, compared to that of the wild type, we found the fitness of a Δ*graR* mutant plated on these antibiotics to be reduced by 4 to 5 orders of magnitude (Fig. 2B). This mutant was also sensitive to ciprofloxacin, although less so than to the other antibiotics tested. The *graXRS/vraFG* regulon includes other global regulators such as *agr* and *walKR*, and its function has been linked to numerous stress response and virulence genes (68). However, its best-characterized role is that of regulation of *mprF* and the *dlt* operon, both of which are involved in modulating cell surface charge. The *dlt* operon attaches d-alanine to lipoteichoic and wall teichoic acids (69), increasing the positive charge of the cell surface. The *dlt* pathway has been shown to modulate resistance to cationic antimicrobial peptides, aminoglycosides, and other positively charged antibiotics (70). Whereas *mprF* was identified as a top hit under several treatment conditions, transposon insertions in the *dlt* genes were poorly represented in the control libraries, because *dlt* mutants exhibit substantial fitness defects even in the absence of antibiotic selection and so do not compete well against the other mutants in the library. However, upon direct testing, we found the fitness of a *dltA* mutant plated on three of the six tested antibiotics—vancomycin, ciprofloxacin, and gentamicin—to be greatly reduced compared to that of the wild type (Fig. 2B). Binding of the zwitterionic fluoroquinolones to the cell surface is known to be antagonized by calcium or magnesium ions, and perhaps the presence of d-alanylation of cell wall components is similarly antagonistic (71). The sensitivity of *dltA* mutants to vancomycin has been previously reported and was suggested to be due to increased binding of vancomycin to the cell surface (72). It was previously shown that the number of positive charges on aminoglycosides correlates with activity against the *dltA* mutant (73), but in our tests, we did not observe a strong correlation between the number of positive charges and the fitness of the *dltA* mutant for different classes of antibiotics. As d-alanylation of the cell envelope results in pleiotropic effects, the fitness of the *dltA* mutant in the presence of different antibiotics likely reflects its different cellular roles. Nevertheless, our results suggest that the importance of the *graRS/vraFG* MCS can be explained in part by the combined action of two members of its regulon, *dltA* and *mprF*, which modify cell envelope charge.

### Two previously uncharacterized genes are broadly important under cell envelope stress conditions.

Among the novel genes identified as hits under one or more sets of treatment conditions, two genes, *SAOUHSC_01025* and *SAOUHSC_01050*, encoding polytopic membrane proteins, stood out as particularly important because both were identified as hits for three of the six antibiotics: oxacillin, vancomycin, and daptomycin. These genes are conserved in *S. aureus*. SAOUHSC_01025 is predicted to have 10 transmembrane domains, with a 93-amino-acid extracellular domain between helices six and seven, while SAOUHSC_01050 is predicted to have 3 transmembrane domains and a 191-amino-acid C-terminal extracellular domain. BLAST and PSI-BLAST searches performed with SAOUHSC_01025 and SAOUHSC_01050 did not reveal extensive amino acid identity with proteins from any other source or of any known function.

**FIG 3:**
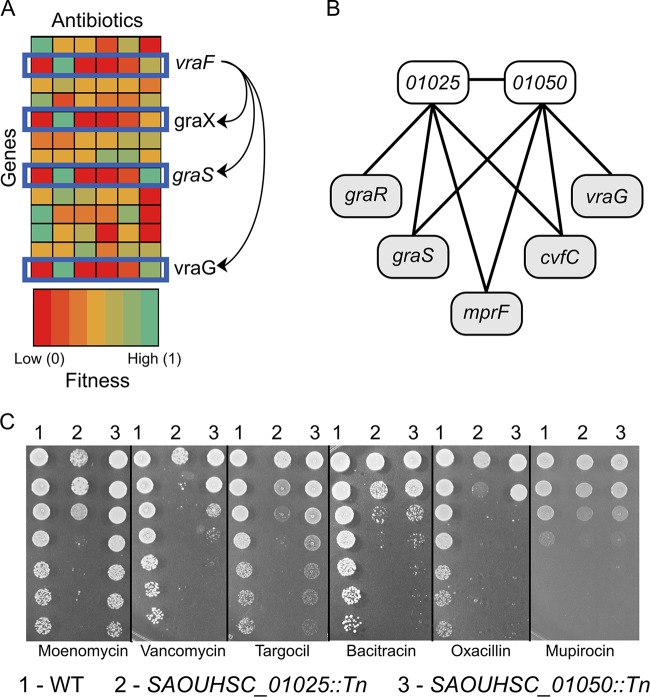
Two genes encoding polytopic membrane proteins were found to be important for withstanding antibiotics that target the cell envelope. (A) Schematic depicting the fitness of a subset of genes upon treatment with different antibiotics. Each column represents an antibiotic, and each row represents a gene. Genes with related functions, such as the components of the *graRS*-*vraFG* MCS, have similar fitness profiles across a panel of antibiotics. Therefore, for any given test gene, it is possible to identify the five genes with the most similar fitness profiles, and it is inferred that these genes are involved in pathways related to the test gene. (B) The K-nearest neighbors algorithm predicted that *SAOUHSC_01025* and *SAOUHSC_01050*, which encode polytopic membrane proteins of unknown function, were most similar to one another and also shared similarity with three of four other identified neighbors. As these neighbors play an important role in protecting *S. aureus* from certain classes of antibiotics, we predict that *SAOUHSC_01025* and *SAOUHSC_01050* are important for cell envelope integrity. (C) Spot dilution assays showing fitness of inactivation mutants in *SAOUHSC_01025* and *SAOUHSC_01050* upon plating on the indicated antibiotics compared to that of the WT (for data representing additional antibiotics, see Fig. S2 in the supplemental material). The first five antibiotics target the cell envelope, and at least one of the two mutant strains is highly sensitive at an antibiotic concentration that permits growth of the WT at all dilutions. The sixth antibiotic targets protein translation, and the mutants show a decrease in fitness of only 1 log compared to that of the WT.

Taking advantage of the data generated via the Tn-Seq experiments, we used a machine learning approach to identify the genes with the fitness profiles (i.e., representing fitness of an inactivation insertion mutation in that gene under each set of antibiotic conditions) most similar to those of *SAOUHSC_01025* and *SAOUHSC_01050* (see Materials and Methods). We first tested the K-nearest neighbors algorithm by using *graX*, *graS*, *vraF*, or *vraG* as the query gene and then searched across all nonessential genes for those with the most similar fitness profiles for all six antibiotic selections. For each of these genes, at least two of the other components of the *graXRS/vraFG* MCS system were among the five genes that most closely paralleled their contribution to fitness in these tests. This result was expected because the individual components of the *graXRS/vraFG* system would be expected to exhibit similar fitness profiles (Fig. 3A). We then applied the algorithm to identify the five genes with the profiles most similar to those of mutants with insertions in *SAOUHSC_01025* and *SAOUHSC_01050*. Transposon mutations in *SAOUHSC_01025* and *SAOUHSC_01050* were most similar in their effect to one another, and they shared three of four additional nearest neighbors, *graS*, *mprF*, and *cvfC* (Fig. 3B). In addition, *graR* and *vraG* were identified as similar to *SAOUHSC_01025* and *SAOUHSC_01050*, respectively*.* These data collectively indicate that SAOUHSC_01025 and SAOUHSC_01050 are new factors involved in maintaining membrane integrity and withstanding envelope stress.

Using the agar spot dilution assay, we tested the fitness of mutants with transposon insertions in *SAOUHSC_01025* (*SAOUHSC_01025*::*Tn*) and *SAOUHSC_01050* (*SAOUHSC_01050*::*Tn*) against a panel of 12 antibiotics with a greater range of different targets. In addition to the six antibiotics originally used, we added moenomycin, targocil, bacitracin, fosfomycin, mupirocin, and rifampin (Fig. 3C; see also Fig. S2 in the supplemental material). Moenomycin inhibits peptidoglycan synthesis by binding to the extracellular transglycosylases that polymerize lipid II (74–76), while bacitracin inhibits the same pathway by binding to undecaprenylpyrophosphate released during lipid II polymerization, thereby preventing lipid II recycling and new lipid II synthesis (77). Fosfomycin inhibits peptidoglycan synthesis by inhibiting an intracellular enzyme, MurA (78). Targocil inhibits wall teichoic acid biosynthesis, resulting in depletion of peptidoglycan precursors and, therefore, inhibition of peptidoglycan synthesis (36, 79, 80). Mupirocin inhibits protein translation by targeting an acyl-tRNA synthetase (81), and rifampin inhibits RNA polymerase (82, 83). Against cell wall-active antibiotics, *SAOUHSC_01025*::*Tn* and *SAOUHSC_01050*::*Tn* generally showed large reductions in fitness, although *SAOUHSC_01025*::*Tn* was typically more susceptible than *SAOUHSC_01050*::*Tn.* Moenomycin provides a striking example of this: the fitness of the *SAOUHSC_01025*::*Tn* mutant decreased by 4 orders of magnitude whereas the fitness of the *SAOUHSC_01050*::*Tn* mutants did not change. Both the *SAOUHSC_01025*::*Tn* and *SAOUHSC_01050*::*Tn* mutants plated on non-cell wall-related antibiotics showed a more modest reduction in fitness. The one exception was that *SAOUHSC_01050*::*Tn* was found to be far more sensitive to gentamicin than *SAOUHSC_01025*::*Tn*, a unique behavior of *SAOUHSC*_*01050*::*Tn* not observed with any other antibiotic. The greatly decreased fitness of both *SAOUHSC_01025*::*Tn* and *SAOUHSC_01050*::*Tn* mutants with a wide range of cell wall-active antibiotics verifies that they play an important role in cell envelope integrity.

There are a number of ways in which *SAOUHSC_01025* and *SAOUHSC_01050* could affect cell envelope integrity. Because these genes have fitness profiles similar to those of *graRS/vraFG* and *mprF*, it is tempting to suggest that their expression is regulated by this MCS. However, this system has been very well studied, and these two genes have not been identified as members of the *graRS/vraFG* regulon (68, 84). Another possibility is that these genes are part of a parallel cell envelope stress pathway, responding to stresses similar to those responded to by the *graRS/vraFG* MCS. Both genes are predicted to encode proteins with extracellular domains. It is possible that these extracellular domains could serve a variety of functions, including acting as sensory domains which respond to a small molecule or other metabolites produced as a result of cell envelope stress. The possibility that these proteins could be acting as scaffolding proteins, coordinating cell envelope synthesis or repair, cannot be ruled out. The heightened sensitivity of *SAOUHSC_01050*::*Tn* to gentamicin suggests that this protein plays a role, directly or indirectly, in regulating cell membrane potential or cell envelope positive charge. Future work will provide insight into the cellular functions of these highly important intrinsic resistance factors.

### Conclusion.

Treating transposon libraries with subinhibitory concentrations of antibiotics using a format that involves massively parallel competition between mutants allows the robust identification of factors that contribute to resistance. Mutant fitness based on Tn-seq profiles correlated well among multiple approaches, including assessment of fitness of individual mutants plated on antibiotics. Previously characterized intrinsic resistance factors were identified, validating the method, but the use of saturating mutant pools in a massively parallel assay also identified novel intrinsic resistance factors, including those contributing to resistance under conditions of treatment with antibiotics in multiple classes. We also used a machine learning approach to obtain insights into the physiological roles of genes annotated as hypothetical. Two novel intrinsic resistance factors, *SAOUHSC_01025* and *SAOUHSC_01050*, were found to be important in withstanding cell envelope damage, but elucidation of the mechanism by which they do so will require further direct characterization. These or other intrinsic resistance factors may be of considerable value as targets for the development of small-molecule potentiators that extend the utility of existing and new antibiotics for treating *S. aureus* infections.

## MATERIALS AND METHODS

### Library 1 antibiotic treatment and DNA preparation.

Library 1 was constructed by transformation of a temperature-sensitive plasmid as previously described (26). Briefly, a 100-µl aliquot of this initial *S. aureus* HG003 transposon library freezer stock, containing 10^8^ CFU, was used to inoculate 100 ml of Mueller-Hinton (MH) cation-adjusted broth and incubated for 15 h at 37°C with shaking at 200 rpm. A 10-µl aliquot (10^6^ CFU) of this input culture was then inoculated into a final volume of 200 µl in a 96-well plate broth microdilution format and incubated at 37°C for 8 h, representing approximately 5.5 generations (5 × 10^7^ CFU/200 µl). The 0.5×, 0.25×, and 0.125× MIC wells for the library pool were determined on the basis of the MIC of a small mutant pool (consisting of 10 innocuous transposon mutants). This small pool was used to determine MICs in order to compensate for resistant mutants potentially present in the library pool. The chosen wells (0.5×, 0.25×, and 0.125×) were then subcultured (3 × 10^5^ CFU) into a second iteration of serial dilutions of antibiotics as described above and incubated for 15 h at 37°C, representing approximately 9 generations (2 × 10^8^ CFU/200 µl). The 0.5×, 0.25×, and 0.125× MIC wells were determined based on the small pool, and these wells were transferred to 10 ml of brain heart infusion (BHI) broth and incubated for 4 h at 37°C with shaking at 200 rpm. Biological replicates were conducted for each growth condition. Genomic DNA was harvested using a DNeasy blood and tissue kit (Qiagen, Valencia, CA) following the manufacturer’s instructions. Library 1 was prepared for next-generation sequencing using the shearing method as described previously (27).

### Library 2 antibiotic treatment and DNA preparation.

Library 2 was constructed by phage-based transposition of six different transposon constructs as previously described (14). Tryptic soy broth (TSB) supplemented with 25 mg/liter Ca^2+^ and 12.5 mg/liter Mg^2+^ was used for all antibiotics except oxacillin, which was tested using cation-adjusted Mueller-Hinton broth (MHB). For all antibiotics, an untreated control was prepared in the same media as was used for the tested antibiotic. A stock of the complete library was thawed and diluted to an optical density at 600 nm (OD_600_) of 0.2 and grown to an OD_600_ of ~0.4 to minimize changes in library composition prior to treatment. The culture was then diluted to 4 × 10^5^ CFU/ml and added to 1 ml of media with 2× the desired concentration of the antibiotic to give a final starting inoculum of 2 × 10^5^ CFU/ml in 2-ml culture volumes. A 10^7^ CFU/2 ml starting inoculum was used for vancomycin- and daptomycin-treated samples. Samples were grown at 37°C and harvested when they reached stationary phase (~1 × 10^9^ CFU/2 ml). The samples were treated with 2×, 1×, 0.5×, and 0.25× the MIC of the antibiotic. Then, we identified antibiotic concentrations that caused the transposon library to reach stationary phase with a few hours of delay compared to the untreated control. These antibiotic concentrations were prepared for sequencing following the protocol described by Santiago et al. (14). Samples from at least two of the concentrations of library 2 were sent for sequencing. Illumina sequencing was completed at the Harvard Biopolymers Facility or the Tufts Genomic DNA Sequencing Core Facility on a HiSeq 2500 sequencing system.

### Data analysis of both libraries.

We identified datasets from library 2 where reads mapped to approximately 25% to 40% of the TA dinucleotide sites with hits in the untreated control (with the exception of the vancomycin treatment, which hit 67% of the TA dinucleotide sites hit in the untreated control). These were processed for further analysis. This percentage of decrease was chosen so that we could identify genes with an increase and a decrease in the number of reads mapping to them. Library 2 contains transposon constructs with outward-facing promoters that can upregulate proximal genes in addition to the traditional construct which can only insert into and inactivate genes. For these experiments, we considered only data from the inactivation constructs. Data for both library 1 and library 2 were analyzed as described previously (14), with the following modifications using in-house python scripts. Data from biological replicates were combined, and before the numbers of reads/gene were compared using the Mann-Whitney *U* test, the experimental data were normalized to the control data using simulation-based resampling (28, 29). Data for each antibiotic treatment from each of the library 1 experiments were then combined with the data from the library 2 experiments using the geometric mean of the ratios of reads in the antibiotic-treated sample compared to the control and Fisher’s method for combining corrected *P* values. Top hits were identified first by filtering for genes with a *P* value of less than 0.05 and then by increasing the fold change cutoff value by integers until 20 genes or fewer were left.

### Spot dilution assays.

Identified hits *qoxA*, *qoxB*, *ndh*, *fmtA*, *SAOUHSC_01025*, and *SAOUHSC_01050* were validated using transposon mutants from the Nebraska library in the USA300 LAC JE2 background (58). *dltA* and *graR* deletion mutants were tested in methicillin-susceptible *S. aureus* (MRSA) strain Newman (17). Agar plates were prepared with TSB supplemented with Ca^2+^ (25 mg/liter) and Mg^2+^ (12.5 mg/liter) and the six antibiotics at concentrations below the MIC. Overnight cultures of mutants were diluted 1:100 in fresh TSB and grown to an OD_600_ of 1. They were then serially diluted 10-fold, spotted onto an agar plate, and incubated at 37°C overnight. The concentration of the antibiotic at which growth of the WT was severely inhibited, showing growth in only the highest 1 or 2 dilutions on agar plates under these conditions, was determined. This was considered to be the MIC under these conditions. The spot dilution assays were then set up using three different antibiotic concentrations. The concentration closest to the MIC at which the WT was at most 3 logs more depleted than the control was used to calculate fitness. This concentration was used so that reduced fitness of any mutants could be observed. The exception to this was the use of the MIC for gentamicin to evaluate the resistance of inactivation mutants in the oxidative phosphorylation pathway (see Fig. S1 in the supplemental material). Control plates with no antibiotic were set up for all strains assayed, and under these conditions, the mutant and WT strains showed equal levels of growth (not shown). Fitness was assessed by determining the highest dilution for which growth was observed for a mutant and the WT strain. The highest dilution showing full growth for the mutant was then divided by the highest dilution showing full growth for the WT to calculate the fitness of the mutant compared to that of the WT, and the results were plotted on a log scale. Those spots that showed hazy growth indicative of cell lysis, those that showed mixed populations of colonies of different sizes that suggested the possible presence of suppressors and reduced fitness relative to spots with homogenous colonies, and those that had fewer than 10 individual colonies were regarded as not representative of full growth.

### Machine learning algorithm optimization.

We used the machine learning algorithm K-nearest nearest neighbors to identify other genes with similar resistance and sensitization patterns in an unsupervised manner using the Scikit-learn python library (85). However, because of the different selective pressures exerted by each antibiotic, the ratio of reads under the experimental treatment conditions versus the control conditions that map to each gene could not be used as the metric for classification. In addition, we wanted to distinguish between the two following conditions: (i) a ratio change of 0.1 due to 100 reads in the control and 10 reads in the experiment and (ii) a ratio change of 0.1 due to 1,000 reads in the control and 100 reads in the experiment. For two genes of same length, option 1 is much less relevant than option 2, as 100 reads/gene and 10 reads/gene both correspond to a gene with a significant fitness defect whereas a change from 1,000 reads/gene to 100 reads/gene is more likely to be a significant change. Therefore, we converted the ratios to a more appropriate fitness measurement value by first choosing a value for the minimum number of reads per gene that could be considered interesting. Based on empirical observations, we noticed that essential/fitness-defective genes tended to have <1/10,000 of the total number of reads in the sample, so the value for any gene with fewer reads mapping to it was converted to this value. Then, ratios were recalculated. Next, the new modified ratio was multiplied by the number of reads mapping to that gene under the treatment conditions and was normalized to the length of the gene. Genes were ordered from lowest to highest “fitness” level. To place all the samples on the same scale, the gene with the lowest “fitness” was given a value of 0, and the gene with the highest “fitness” was given a value of 1. All other genes were placed in order between these values, in increments that increased by 1/(total number of genes). This final value, which we call the “normalized fitness value,” was subsequently used in the machine learning analysis. Essential genes were removed to reduce bias in the data set, and the K-nearest neighbors algorithm was further optimized by adjusting the Minkowski distance metric to output the genes with the resistance/sensitization patterns most similar to those of the test gene. We identified the five genes (the five nearest neighbors) with the most similar patterns of “normalized fitness values.”

### Data availability.

All raw next-generation sequencing data as well as the python scripts used in the analysis are available on the publically accessible Harvard Dataverse Network at https://dataverse.harvard.edu/dataverse/intrinsicresistancefactordata.

## SUPPLEMENTAL MATERIAL

Figure S1  Inactivation of the oxidative phosphorylation pathway confers resistance to gentamicin. (A) Schematic of the oxidative phosphorylation pathway is depicted here. Numbers of reads due to transposon insertions were greatly increased under conditions of exposure to gentamicin for the 11 genes illustrated. Inactivation of the oxidative phosphorylation pathway is a known mechanism of resistance to gentamicin, which depends on the membrane potential for cell entry. (B) Results representative of the fold change in the number of reads/gene for a subset of genes involved in oxidative phosphorylation that were tested to determine if inactivation confers resistance to gentamicin. (C) Fitness comparison of the WT to mutant strains in which the indicated genes were inactivated. Spot dilutions of WT and mutant strains were plated on gentamicin, and fitness was calculated as the ratio of the highest dilution that allowed growth of the WT to highest dilution that allowed growth of the mutant (see Materials and Methods). Download Figure S1, PDF file, 0.2 MB

Figure S2  *SAOUHSC_01025*::*Tn* and *SAOUHSC_01050*::*Tn* are particularly sensitive to antibiotics that damage the cell envelope. (A) Data representative of results of analysis of the targeted pathways of the additional antibiotics tested against *SAOUHSC_01025*::*Tn* and *SAOUHSC_01050*::*Tn* mutants are shown. The abbreviations used here are as follows: mup, mupirocin; lin, linezolid; rif, rifampin; cip, ciprofloxacin; gen, gentamicin; van, vancomycin; bac, bacitracin; tar, targocil; fos, fosfomycin; moe, moenomycin A; dap, daptomycin. (B) A summary of the fitness of these mutants relative to that of the WT was assessed by spot dilution against the various antibiotics tested. (C) Spot dilution assay plates for these mutants and all antibiotics tested are shown here. The results obtained with the first six antibiotics are reproduced from Fig. 3 for comparison. Download Figure S2, PDF file, 0.3 MB

Table S1  A total of 80 unique genes were identified as important for fitness by treatment of pooled transposon libraries with six antibiotics. The top 20 genes with the greatest fold change in numbers of mapped reads are shown for each antibiotic. Fold change in the number of mapped reads is indicated by colored rectangles. Orange rectangles indicate genes for which the numbers of reads due to transposon insertions were substantially lower than in the control, whereas blue rectangles indicate genes for which the numbers of reads due to transposon insertions were substantially higher than in the control. Gray rectangles indicate that they were not identified as a hit using that antibiotic treatment. Table S1, PDF file, 0.3 MB
