# Supplementary figures and images for: Multidrug Intrinsic Resistance Factors in Staphylococcus aureus Identified by Profiling Fitness within High-Diversity Transposon Libraries
Source: mBio. 2016 Aug 16;7(4):e00950-16. doi: 10.1128/mBio.00950-16 (PMC4992970; doi:10.1128/mBio.00950-16)

A

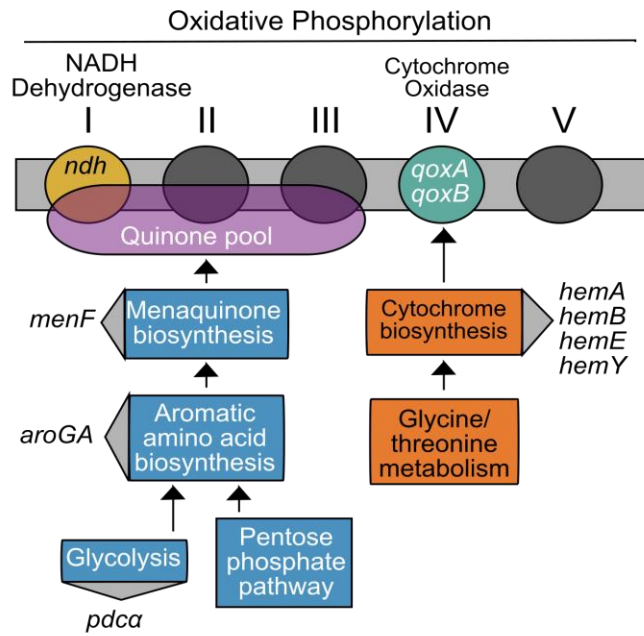

B

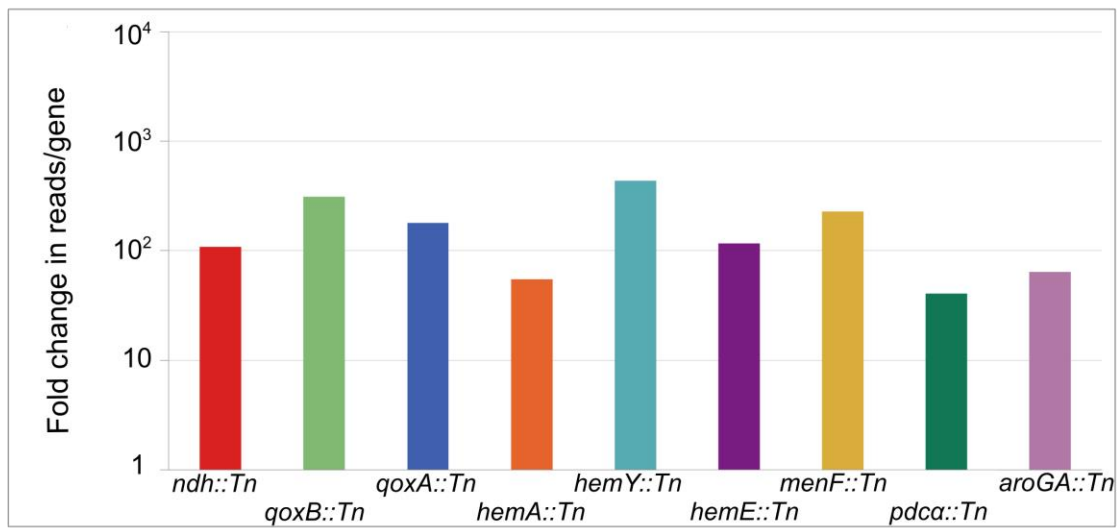

C

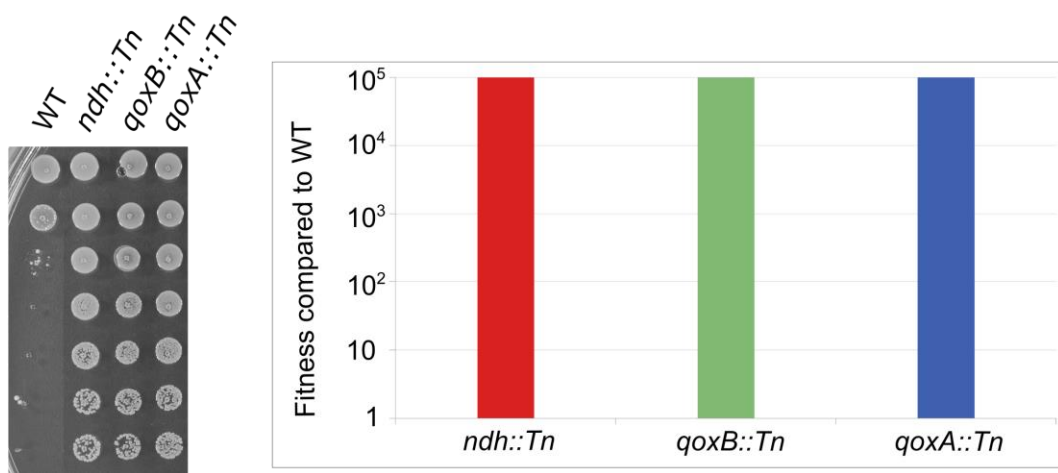

Supplement: Figure S1 — Inactivation of the oxidative phosphorylation pathway confers resistance to gentamicin. (A) Schematic of the oxidative phosphorylation pathway is depicted here. Numbers of reads due to transposon insertions were greatly increased under conditions of exposure to gentamicin for the 11 genes illustrated. Inactivation of the oxidative phosphorylation pathway is a known mechanism of resistance to gentamicin, which depends on the membrane potential for cell entry. (B) Results representative of the fold change in the number of reads/gene for a subset of genes involved in oxidative phosphorylation that were tested to determine if inactivation confers resistance to gentamicin. (C) Fitness comparison of the WT to mutant strains in which the indicated genes were inactivated. Spot dilutions of WT and mutant strains were plated on gentamicin, and fitness was calculated as the ratio of the highest dilution that allowed growth of the WT to highest dilution that allowed growth of the mutant (see Materials and Methods). Download [file mbo004162934sf1.pdf]
