# Supplementary material for: Multidrug Intrinsic Resistance Factors in Staphylococcus aureus Identified by Profiling Fitness within High-Diversity Transposon Libraries
Source: mBio. 2016 Aug 16;7(4):e00950-16. doi: 10.1128/mBio.00950-16 (PMC4992970; doi:10.1128/mBio.00950-16)

A

Antibiotic Target	Antibiotics
Cell Wall (PG)	Fosfomycin, Bacitracin, MoenomycinA, Oxacillin, Vancomycin
Cell Membrane	Daptomycin
Wall Teichoic Acid Synthesis	Targocil
DNA Gyrase/Topoisomerase	Ciprofloxacin
Ribosome	Gentamicin, Linezolid, Mupirocin
RNA Synthesis	Rifampicin

B

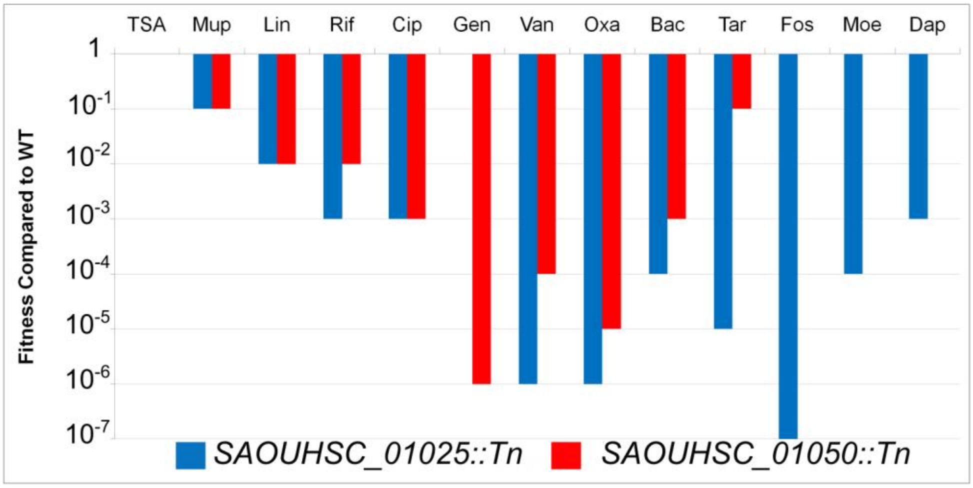

C

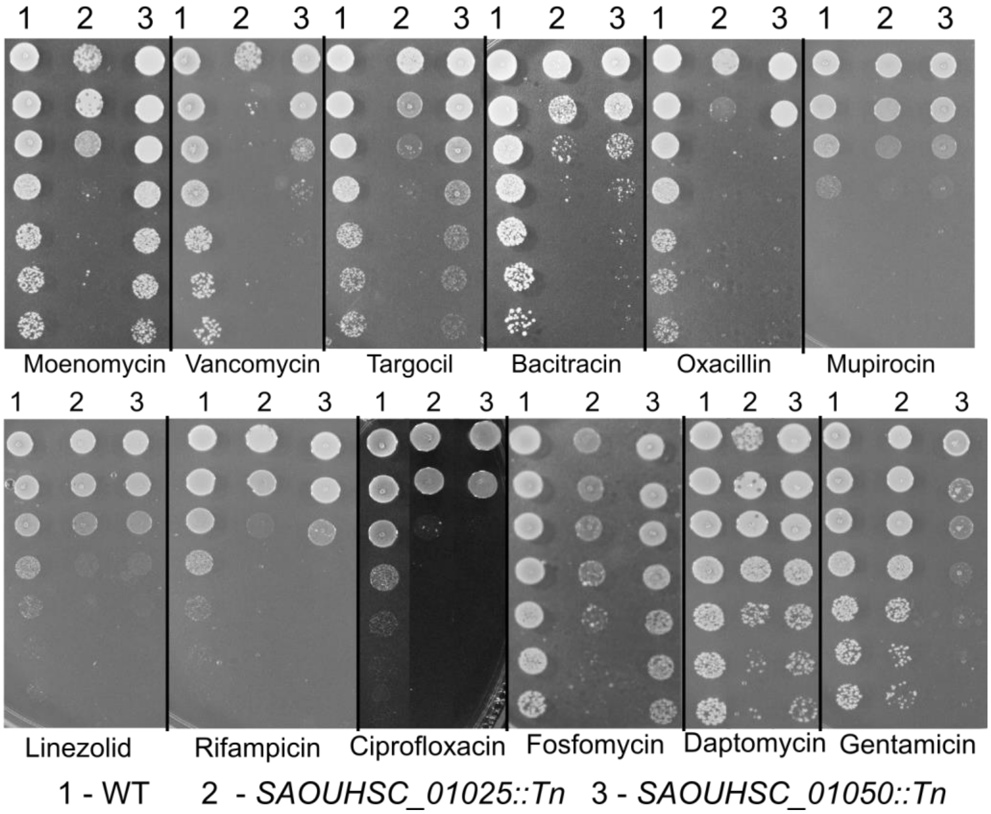

Supplement: Figure S2 — SAOUHSC_01025::Tn and SAOUHSC_01050::Tn are particularly sensitive to antibiotics that damage the cell envelope. (A) Data representative of results of analysis of the targeted pathways of the additional antibiotics tested against SAOUHSC_01025::Tn and SAOUHSC_01050::Tn mutants are shown. The abbreviations used here are as follows: mup, mupirocin; lin, linezolid; rif, rifampin; cip, ciprofloxacin; gen, gentamicin; van, vancomycin; bac, bacitracin; tar, targocil; fos, fosfomycin; moe, moenomycin A; dap, daptomycin. (B) A summary of the fitness of these mutants relative to that of the WT was assessed by spot dilution against the various antibiotics tested. (C) Spot dilution assay plates for these mutants and all antibiotics tested are shown here. The results obtained with the first six antibiotics are reproduced from Fig. 3 for comparison. Download [file mbo004162934sf2.pdf]
