# Supplementary material for: Multidrug Intrinsic Resistance Factors in Staphylococcus aureus Identified by Profiling Fitness within High-Diversity Transposon Libraries
Source: mBio. 2016 Aug 16;7(4):e00950-16. doi: 10.1128/mBio.00950-16 (PMC4992970; doi:10.1128/mBio.00950-16)
Supplement: Table S1 — A total of 80 unique genes were identified as important for fitness by treatment of pooled transposon libraries with six antibiotics. The top 20 genes with the greatest fold change in numbers of mapped reads are shown for each antibiotic. Fold change in the number of mapped reads is indicated by colored rectangles. Orange rectangles indicate genes for which the numbers of reads due to transposon insertions were substantially lower than in the control, whereas blue rectangles indicate genes for which the numbers of reads due to transposon insertions were substantially higher than in the control. Gray rectangles indicate that they were not identified as a hit using that antibiotic treatment. [file mbo004162934st1.pdf]

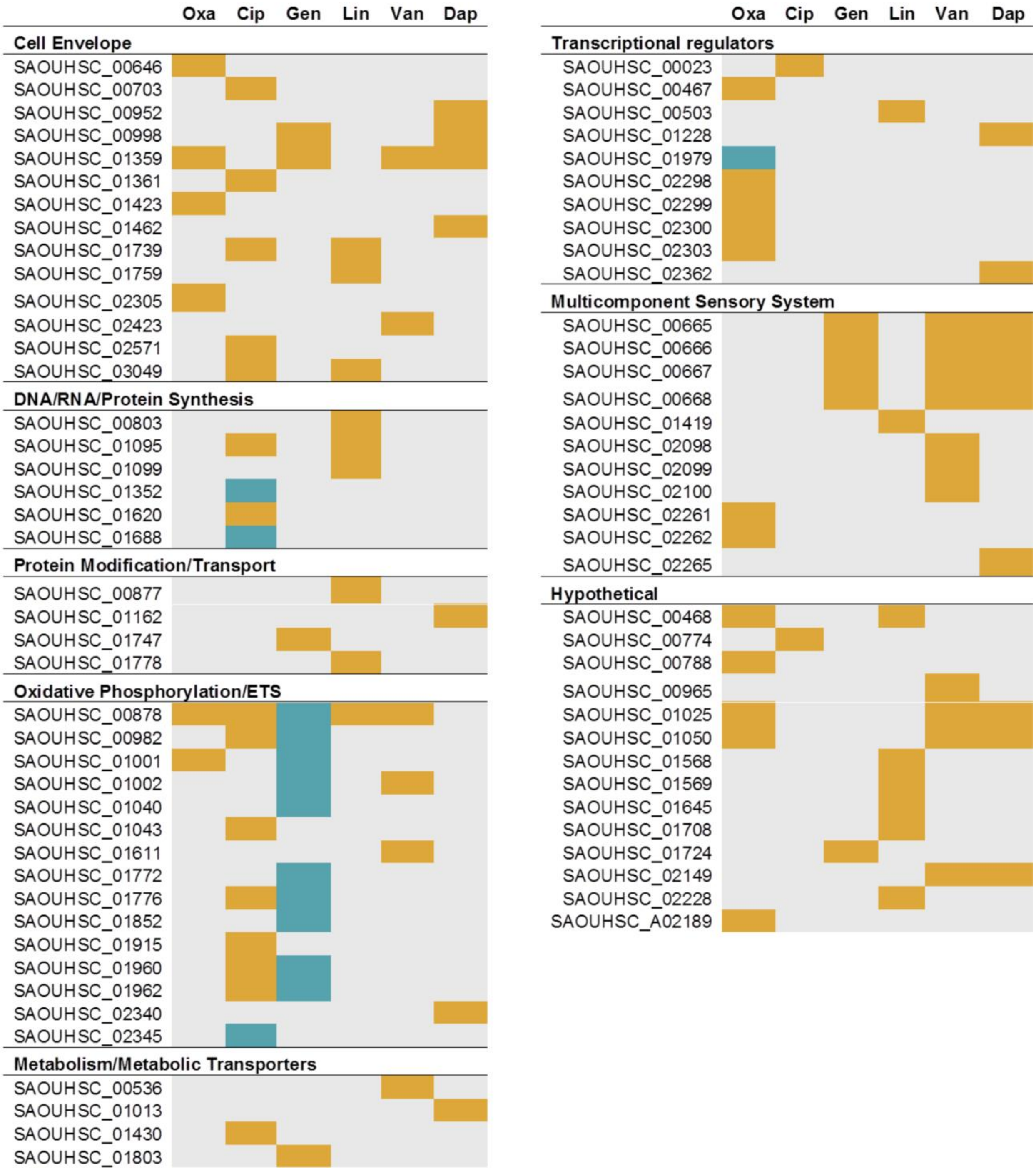
Oxa
Cip
Gen
Lin
Van
Dap
Cell Envelope
SAOUHSC_00646
SAOUHSC_00703
SAOUHSC_00952
SAOUHSC_00998
SAOUHSC_01359
SAOUHSC_01361
SAOUHSC_01423
SAOUHSC_01462
SAOUHSC_01739
SAOUHSC_01759
SAOUHSC_02305
SAOUHSC_02423
SAOUHSC_02571
SAOUHSC_03049
DNA/RNA/Protein Synthesis
SAOUHSC_00803
SAOUHSC_01095
SAOUHSC_01099
SAOUHSC_01352
SAOUHSC_01620
SAOUHSC_01688
Protein Modification/Transport
SAOUHSC_00877
SAOUHSC_01162
SAOUHSC_01747
SAOUHSC_01778
Oxidative Phosphorylation/ETS
SAOUHSC_00878
SAOUHSC_00982
SAOUHSC_01001
SAOUHSC_01002
SAOUHSC_01040
SAOUHSC_01043
SAOUHSC_01611
SAOUHSC_01772
SAOUHSC_01776
SAOUHSC_01852
SAOUHSC_01915
SAOUHSC_01960
SAOUHSC_01962
SAOUHSC_02340
SAOUHSC_02345
Metabolism/Metabolic Transporters
SAOUHSC_00536
SAOUHSC_01013
SAOUHSC_01430
SAOUHSC_01803
Oxa
Cip
Gen
Lin
Van
Dap
Transcriptional regulators
SAOUHSC_00023
SAOUHSC_00467
SAOUHSC_00503
SAOUHSC_01228
SAOUHSC_01979
SAOUHSC_02298
SAOUHSC_02299
SAOUHSC_02300
SAOUHSC_02303
SAOUHSC_02362
Multicomponent Sensory System
SAOUHSC_00665
SAOUHSC_00666
SAOUHSC_00667
SAOUHSC_00668
SAOUHSC_01419
SAOUHSC_02098
SAOUHSC_02099
SAOUHSC_02100
SAOUHSC_02261
SAOUHSC_02262
SAOUHSC_02265
Hypothetical
SAOUHSC_00468
SAOUHSC_00774
SAOUHSC_00788
SAOUHSC_00965
SAOUHSC_01025
SAOUHSC_01050
SAOUHSC_01568
SAOUHSC_01569
SAOUHSC_01645
SAOUHSC_01708
SAOUHSC_01724
SAOUHSC_02149
SAOUHSC_02228
SAOUHSC_A02189
